# Identification of differentially expressed mRNA and the Hub mRNAs modulated by lncRNA *Meg3* as a competing endogenous RNA in brown adipose tissue of mice on a high-fat diet

**DOI:** 10.1080/21623945.2020.1789283

**Published:** 2020-07-02

**Authors:** Yemin Zhang, Yalin Fu, Yuyang Zheng, Zhongyuan Wen, Changhua Wang

**Affiliations:** a Department of Pathology & Pathophysiology, Wuhan University School of Basic Medical Sciences, Wuhan, China; b Hubei Provincial Key Laboratory of Developmentally Originated Disease, Wuhan, China; c Demonstration Center for Experimental Basic Medicine Education of Wuhan University, Wuhan, Hubei, China; d Department of Endocrinology, Renmin Hospital of Wuhan University, Wuhan, China

**Keywords:** Brown adipose tissue, high-fat diet, lncRNA *Meg3*, competing endogenous RNA, differentially expressed RNA

## Abstract

Obesity is associated with insulin resistance, diabetes, and obesity-related metabolic disorders. Brown adipocytes have emerged as potential targets for the treatment of obesity and obesity-related diseases. However, changes that occur in brown adipose tissue during various stages of high fat diet (HFD)-induced obesity remain poorly understood. The present study aimed to determine the changes occurring in brown adipose tissue during various stages of an HFD by analyzing two microarray expression profiles. A total of 1,337 differentially expressed RNAs (DE RNAs) were identified between the HFD and ND groups, using the limma package in R. The DE RNAs included 1,249 mRNAs, 74 long non coding RNAs (lncRNAs), and 14 pseudogenes. Functional annotation of the DE mRNAs, including GO terms and KEGG pathways were identified using the Database for Annotation, Visualization, and Integrated Discovery. A protein-protein interaction network was constructed using STRING and clusters were obtained through the Molecular Complex Detection plug-in. In the present study, the lncRNA,maternally expressed gene 3 (*Meg3*), was identified as the DE lncRNA with a significant fold change. The network of *Meg3* as a ceRNA was constructed, which demonstrated that *Meg3* modulated five hub DE mRNAs via competitive binding to microRNAs.

## 1. Introduction

A high-fat-diet (HFD) can induce obesity, which is closely associated with insulin resistance, diabetes, and obesity-related metabolic diseases [1–3]. An increasing number of studies have focused on the impacts of an HFD on white adipose tissue, not only because of its known function as an excess energy reserve, but also because of its function of secreting adipokines during metabolic processes [4]. However, another type of adipose tissue, known as brown adipose tissue (BAT), can dissipate excess energy via non-shivering thermogenesis. During this process, uncoupling protein 1 (UCP1), a unique BAT mitochondrial membrane protein, uncouples respiration from ATP synthesis to produce heat, and stimulates high levels of fatty acid oxidation to release energy [5]. BAT was previously thought to exist only during infancy; however, it has now emerged as a potential therapeutic target to treat obesity and obesity-related diseases, since BAT has also been found to exist in the dorsal interscapular region of adult humans [6,7].

Conventionally, RNA can be classified into two types according to its protein-coding potential. The first type is coding RNA, which can be translated into proteins that determine distinct phenotypes and biological functions [8] and the second type is non-coding RNA, which lacks protein-coding ability. Non-coding RNA can be further divided into subtypes, including microRNA (miRNA) and long non-coding RNA (lncRNA). LncRNAs were once considered to be transcriptional noise, because they consist of transcripts exceeding 200 nucleotides in length, but with no protein-coding potential. However, in recent years, accumulating evidence has demonstrated that lncRNAs act as competing endogenous RNAs (ceRNAs), which posttranscriptionally modulate mRNAs by competitively binding to microRNAs using complementary base pairing [9,10].

Several lncRNAs, including maternally expressed gene 3 (*Meg3*), have been identified as ceRNAs implicated in pathophysiological processes of metabolic disorders [11–13]. *Meg3* has been shown to serve as a ceRNA to regulate the *miR-302a-3p*-CRTC2 axis and the *miR-214*-ATF4 axis, and promote hepatic insulin resistance [11,12]. Chen et al. reported that *Meg3*, acts as a ceRNA by directly binding to *miR-145* in cardiomyocytes, thereby upregulating the expression of PDCD4 to induce cardiomyocyte apoptosis under high-glucose conditions [13]. However, changes in *Meg3* levels and its corresponding function in BAT under HFD conditions remain ill-defined.

The previous researches pay close attention to the alteration of thermogenic-related mRNAs and lncRNAs during the differentiation of brown preadipocytes or in the BAT exposing to cold temperature [14,15]. To better understand changes in mRNA levels, the functional enrichment of differentially expressed (DE) mRNAs, and the exact function of lncRNAs as ceRNAs in BAT under HFD conditions, two microarray expression profiles were retrieved from the Gene Expression Omnibus (GEO, http://www.ncbi.nlm.nih.gov/geo/), an international public repository of high-throughput microarray data and relevant functional genomic data sets [16]. Data from BAT samples of male C57BL/6 mice fed an HFD or a normal diet (ND) were collected from two datasets. A total of 1,337 DE RNAs were identified, including 1,249 mRNAs, 74 lncRNAs, and 14 pseudogenes, between the HFD and ND groups, using the limma package in R. Functional annotation of the DE mRNAs, including gene ontology (GO) terms and Kyoto Encyclopaedia of Genes and Genomes (KEGG) pathways, was performed using the Database for Annotation, Visualization, and Integrated Discovery (DAVID). A protein-protein interaction (PPI) network was constructed using the Search Tool for the Retrieval of Interacting Genes (STRING) and clusters were obtained using the Molecular Complex Detection (MCODE) plug-in. The lncRNA, *Meg3*, was identified as the lncRNA with a significant fold change. A network was constructed with *Meg3* as a ceRNA, which demonstrated that *Meg3* modulated several mRNAs by competitively binding to microRNAs. Five hub DE mRNAs were identified in the *Meg3* ceRNA network. These results may deepen our understanding of the BAT function in HFD-induced obesity and obesity-related disorders such as diabetes.

## 2. Materials and methods

### 2.1. Ethical declaration

This research does not directly contain any material obtained from animals or humans. All data used in this study were extracted from public databases.

### 2.2. Microarray data archives

Microarray expression profiles from GSE113315 and GSE116225 datasets were retrieved from the GEO database. Beginning at 3 weeks of age, the male C57BL/6mice were fed regular chow diet (ND, D12450B, Research Diet, Inc. New Brunswick, NJ, USA) or HFD (D12492, Research Diet, Inc. New Brunswick, NJ, USA) ad libitum for 15 weeks [17,18]. ND serves as a negative control. The mice were kept in enclosures at room temperature with a fixed 12 h light/12 h dark cycle. The expression profiles of GSE113315 were based on the GPL13112 (Illumina HiSeq 2000 [Mus musculus]) platform and the expression profiles of GSE116225 were based on the GPL17021 (Illumina HiSeq 2500 [Mus musculus]) platform. The series matrix files of the two datasets were downloaded from GEO to screen for DE RNAs between BAT samples from the HFD and ND groups.

### 2.3. Microarray data and DEG identification

Following annotation of the matrix files from Ensemble ID to gene symbols using http://grch37.ensembl.org/Mus_musculus/Info/Index, the sva package in R (version 3.6.0; University of California, Berkeley, CA, USA) was applied to correct background expression values and normalize the data [19]. DE RNAs, including mRNAs and lncRNAs, with the threshold criteria of adjusted *p* < 0.05 and |log fold change (FC)| > 1 between the HFD and ND group, were screened via the limma package in R [20]. DE RNA biotypes were identified using the online resource at http://grch37.ensembl.org/Mus_musculus/Info/Index. The pheatmap package in R was subsequently used to plot mRNA and lncRNA heatmaps [21].

### 2.4. GO and pathway enrichment analyses

GO and KEGG pathway analyses were performed using DAVID 6.8 (http://david.ncifcrf.gov) [22]. GO is a commonly used bioinformatics tool that provides comprehensive information on the function of individual gene products, based on defined features. GO analysis involves the identification of biological processes (BP), cellular components (CC), and molecular functions (MF). KEGG is a major database used to understand high-level biological functions and utilities. A gene count >2 and *p* < 0.05 were set as the threshold values.

### 2.5. PPI network creation and hub gene identification

A PPI network of DE mRNAs was constructed using STRING 11.0 (https://string-db.org/), with a combined score > 0.9 as the cut-off value [23]. Significant modules in the PPI network were identified using MCODE 1.5.1, a Cytoscape software plug-in [24]. The parameter for DE mRNA clustering and scoring was set as follows: MCODE score ≥ 4, degree cut-off = 2, node score cut-off = 0.2, max depth = 100, and k-score = 2.

### 2.6. Target miRNA prediction of Meg3 and corresponding target mRNA prediction

*Meg3* was selected to be further investigated for two reasons. Firstly, *Meg3* was the DE lncRNA with a significant fold change (Table S1). Secondly, the full sequence of *Meg3* was available in the NONCODE database (http://www.noncode.org). The *Meg3* sequence was imputed into miRDB (http://www.mirdb.org) to obtain the target miRNAs, according to the principle of complementary base pairing. The top five target scores were chosen as the target prediction results. The corresponding target mRNAs of the predicted miRNAs were identified using the Targetscan database (http://www.targetscan.org). From the list of mRNAs in both the Targetscan results and the DE mRNAs, the top 20 mRNAs were selected according to a comprehensive rank of ‘cumulative weighted context++ scores’.

### 2.7. Construction of Meg3 ceRNA regulatory network

Cytoscape software (Cytoscape, 3.7.1) was used to construct the network of *Meg3* as a ceRNA. The network was visualized using the *Meg3*-miRNA-mRNA triple competing relationship module.

### 2.8. Statistical analysis

Statistical analyses of DE RNAs were performed using R, with the threshold criteria of adjusted *p* < 0.05 and |log FC| > 1 between the HFD and ND groups. A gene count > 2 and *p* < 0.05 were set as the thresholds in the GO and KEGG analyses. The parameters for DE mRNA clustering and scoring in the significant module analyses were set as follows: MCODE score ≥ 4, degree cut-off = 2, node score cut-off = 0.2, max depth = 100, and k-score = 2.

## 3. Results

### 3.1. Identification of HFD-induced DE RNAs in BAT

**Figure 1.**
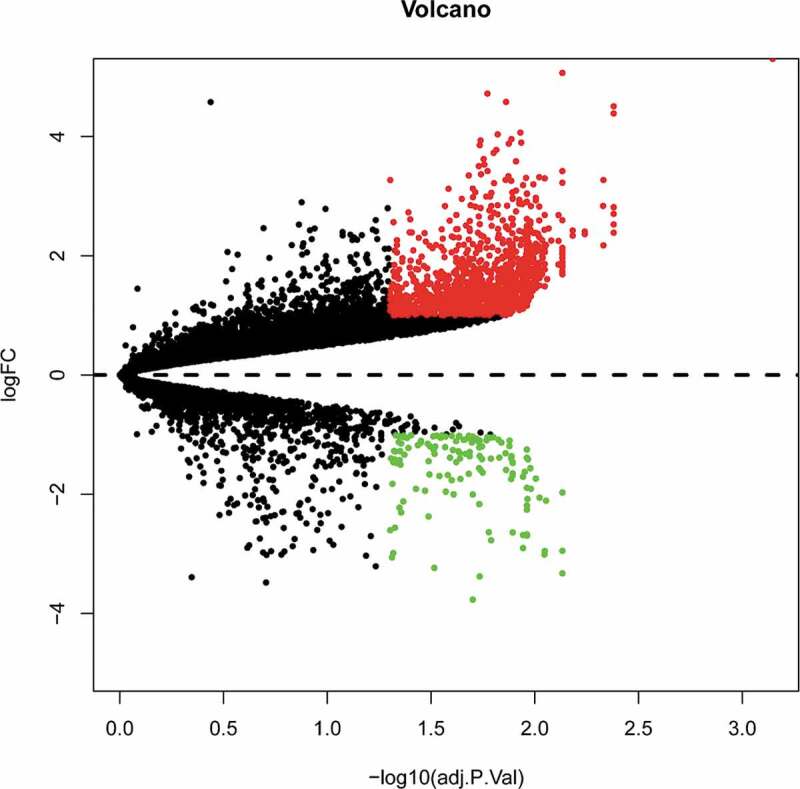
Volcano plot analysis to identify DE RNAs. Red dots represent 1,203 upregulated RNAs and green dots represent 134 downregulated RNAs in BAT from mice fed an HFD or an ND. DE RNA: differentially expressed RNA; HFD: high-fat diet; ND: normal diet; BAT: brown adipose tissue

**Figure 2.**
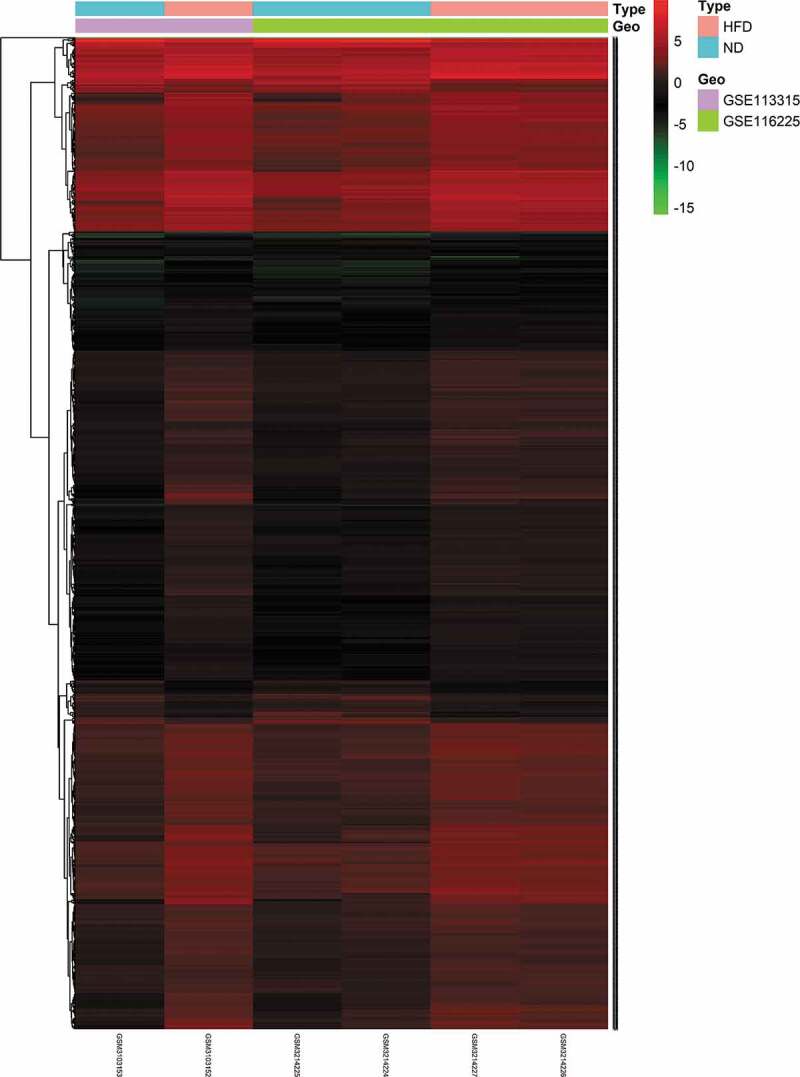
Heatmap of 1,249 DE mRNAs screened using the limma package in R. Red areas represent upregulated genes and green areas represent downregulated genes in BAT from mice fed an HFD compared with mice fed an ND. DE mRNA: differentially expressed mRNA; HFD: high-fat diet; ND: normal diet; BAT: brown adipose tissue

**Figure 3.**
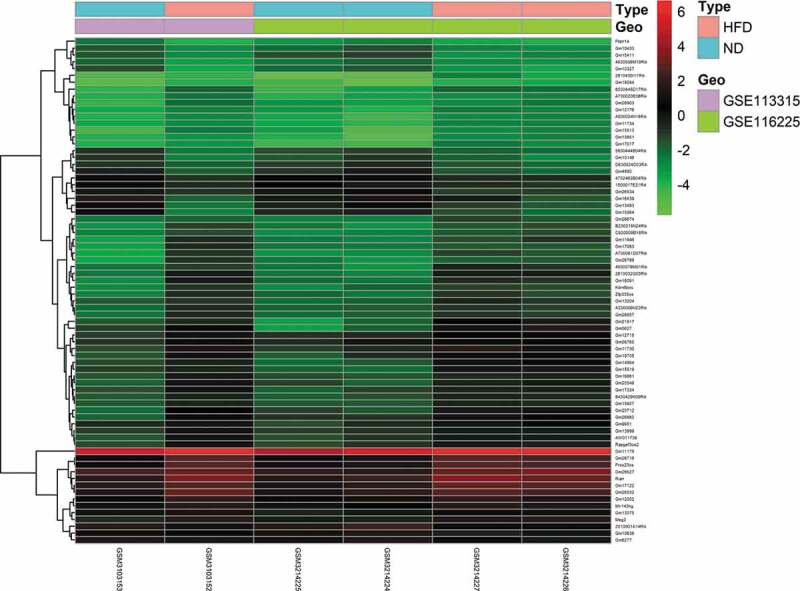
Heatmap of 74 DE lncRNAs screened using the limma package in R. Red areas represent upregulated genes and green areas represent downregulated genes in BAT from mice fed an HFD compared with mice fed an ND. DE lncRNA: differentially expressed lncRNA; HFD: high-fat diet; ND: normal diet; BAT: brown adipose tissue

To identify DE mRNAs and DE lncRNAs in BAT between the HFD and ND groups, we retrieved relevant microarray expression profiles from the GSE113315 and GSE116225 datasets in the GEO database. After the consolidation and normalization of microarray data, 1,337 DE RNAs, including 1,203 upregulated RNAs and 134 downregulated RNAs, were screened using the limma package (|logFC| > 1, adjusted *p *< 0.05) (Figure 1, Table S2). The DE RNA biotypes were identified and found to include 1,249 DE mRNAs, 74 DE lncRNAs, 14 DE pseudogenes between the HFD and ND groups, as shown in the heatmap (Figures 2 and 3).

### 3.2. GO enrichment analysis of DE mRNAs

**Figure 4.**
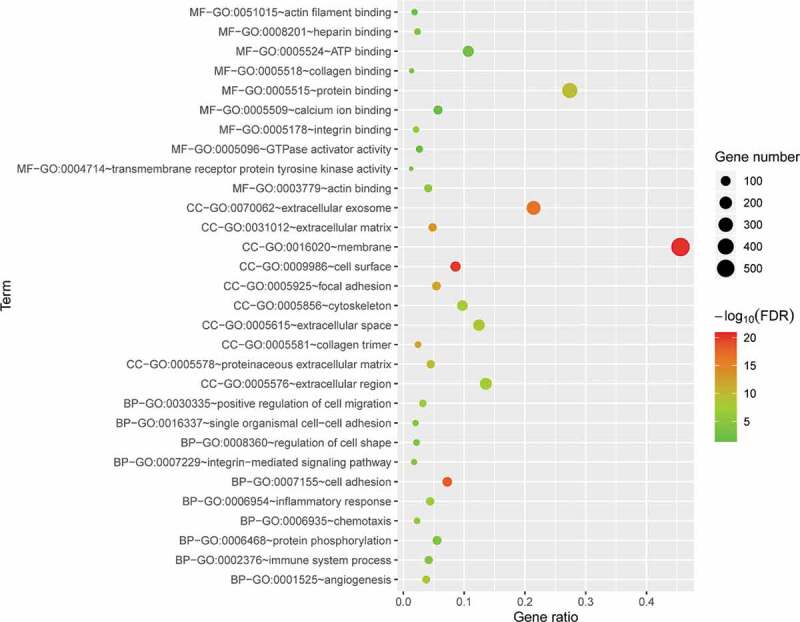
GO enrichment results for DE mRNAs. The x-axis represents the gene ratio and the y-axis represents GO terms. The size of the circle indicates the gene count. Different coloured circles indicate different adjusted *p* values. DE mRNA: differentially expressed mRNA; FDR: false discovery rate; GO: gene ontology

To determine the biological features of the DE mRNAs, GO analysis was performed using the DAVID online tools. The BP analysis showed that the DE mRNAs were mostly enriched for cell adhesion, angiogenesis, and inflammatory response terms (Figure 4). The CC analysis showed that the DE mRNAs were significantly enriched for membrane, cell surface, and extracellular exosome terms (Figure 4) and the MF analysis showed that the DE mRNAs were significantly enriched for protein binding, integrin binding, actin binding, and ATP binding terms (Figure 4).

### 3.3. KEGG enrichment analysis of DE mRNAs

**Figure 5.**
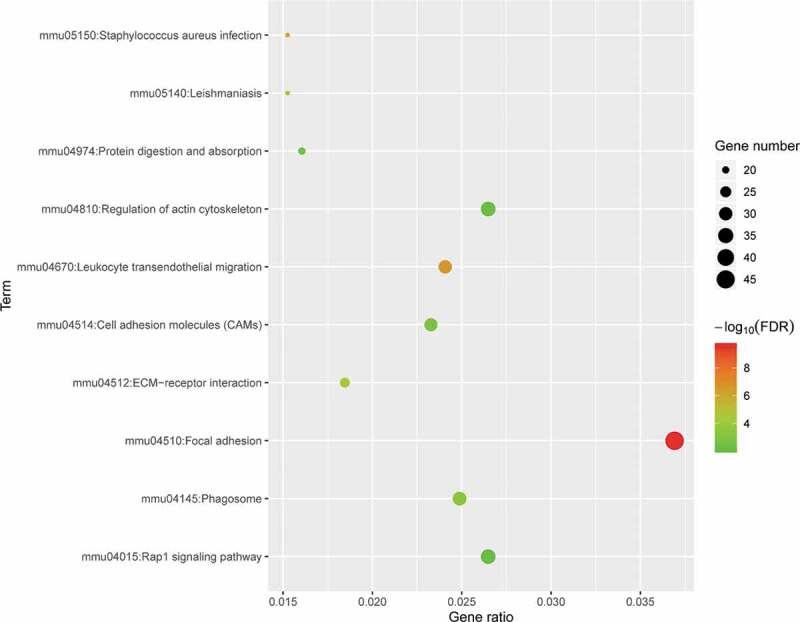
KEGG pathway analysis of differentially expressed genes. Advanced bubble chart shows enrichment of DE mRNAs in signalling pathways. The y-axis represents the pathway and the x-axis represents the rich factor (rich factor = number of DE mRNAs enriched in the pathway/total number of DE mRNAs in the background gene set). The size and colour of the bubble indicates the number of DE mRNAs enriched in the pathway and the enrichment significance, respectively. KEGG: Kyoto Encyclopaedia of Genes and Genomes; DE mRNA: differentially expressed mRNA; FDR: false discovery rate

To explore the potential mechanism responsible for these DE mRNAs, KEGG pathway analysis was performed using DAVID online tools. The results of the KEGG analysis showed that DE mRNAs were mainly involved in focal adhesion, leukocyte transendothelial migration, *Staphylococcus aureus* infection, and ECM-receptor interactions (Figure 5).

### 3.4. PPI network analysis

**Figure 6.**
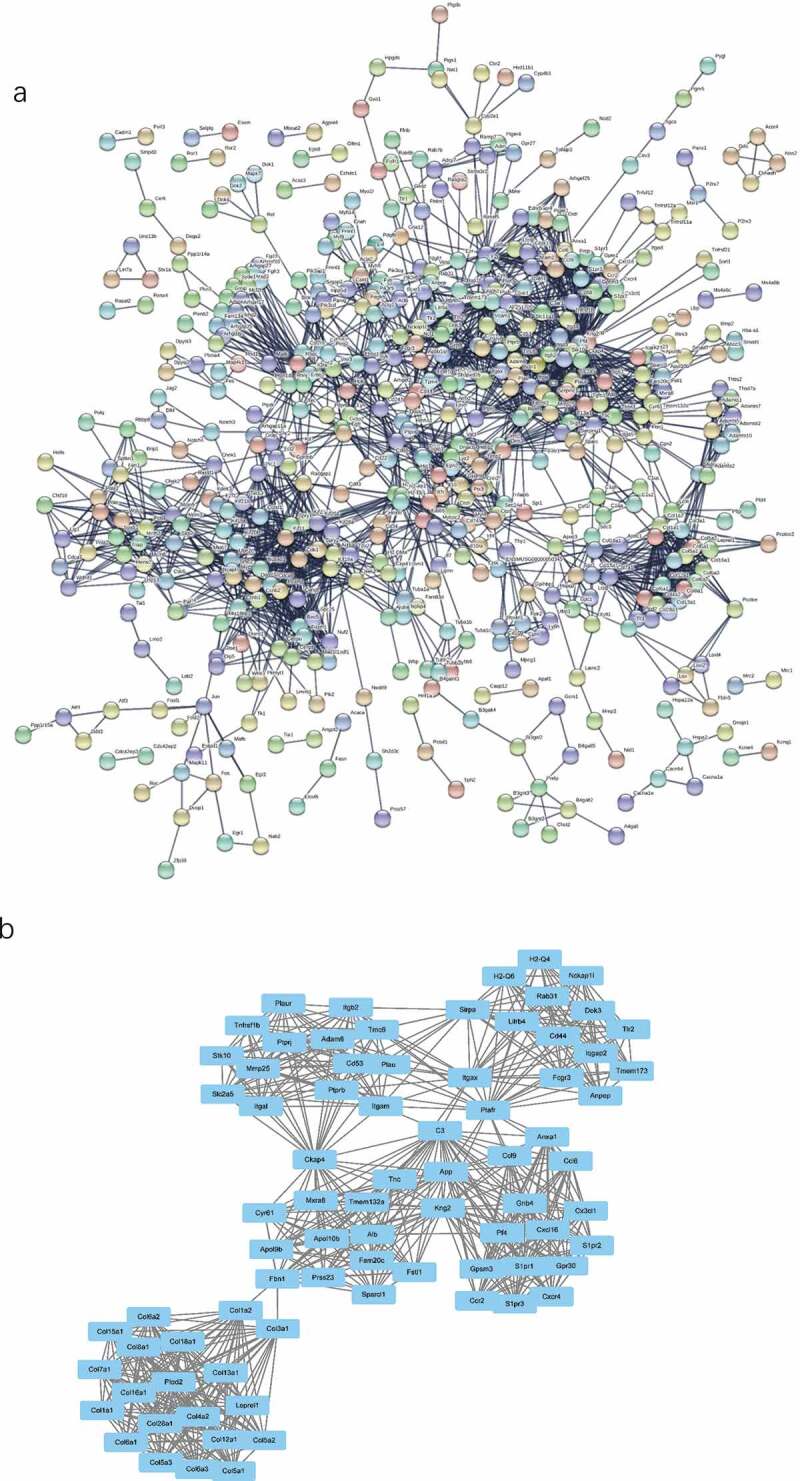
The PPI network and the most significant modules of DE mRNAs. (a) The PPI network was analysed using STRING. There were 1,250 nodes and 2,449 edges in the PPI network. (b) The most significant module identified by MCODE (score = 17.538). DE mRNA: differentially expressed mRNA; PPI: protein-protein interaction

To identify the most significant clusters of the DE mRNAs, a PPI network of DE mRNAs was constructed using STRING. As shown in Figure 6(a), there were 1,238 nodes and 2,429 edges in the PPI network. There were 79 DE mRNAs in the most significant module (score = 17.838) recognized by MCODE (Figure 6(b), Table 1).

**Table 1.** 79 DEmRNAs of the most significant module (score = 17.838) in the BAT between the HFD and ND groups

ID	Logfc	P.Value	adj.P.Val	Gene Name
Adam8	4.066	0.0003	0.0118	A disintegrin and metallopeptidase domain 8
Alb	2.902	0.0002	0.0115	Albumin
Anpep	2.088	0.0025	0.0276	Alanyl (membrane) aminopeptidase
Anxa1	1.947	0.0016	0.0224	Annexin A1
Apol10b	1.174	0.0004	0.0129	Apolipoprotein L 10B
Apol9b	2.302	0.0039	0.0364	Apolipoprotein L 9b
App	1.224	0.0034	0.0341	Amyloid beta (A4) precursor protein
C3	1.275	0.0061	0.0477	Complement component 3
Ccl6	2.238	0.0034	0.0336	Chemokine (C-C motif) ligand 6
Ccl9	2.381	0.0034	0.0341	Chemokine (C-C motif) ligand 9
Ccr2	2.393	0.0046	0.0402	Chemokine (C-C motif) receptor 2
Cd44	2.243	0.0008	0.0170	CD44 molecule (Indian blood group)
Cd53	2.954	0.0017	0.0226	CD53 molecule
Ckap4	1.599	0.0010	0.0183	Cytoskeleton-associated protein 4
Col12a1	2.191	0.0000	0.0090	Collagen, type XII, alpha 1
Col13a1	1.504	0.0003	0.0128	Collagen, type XIII, alpha 1
Col15a1	1.985	0.0005	0.0140	Collagen, type XV, alpha 1
Col16a1	2.483	0.0002	0.0110	Collagen, type XVI, alpha 1
Col18a1	2.121	0.0000	0.0090	Collagen, type XVIII, alpha 1
Col1a1	2.030	0.0002	0.0110	Collagen, type I, alpha 1
Col1a2	1.522	0.0001	0.0108	Collagen, type I, alpha 2
Col28a1	2.057	0.0021	0.0252	Collagen, type XXVIII, alpha 1
Col3a1	1.488	0.0057	0.0459	Collagen, type III, alpha 1
Col4a2	1.069	0.0031	0.0317	Collagen, type IV, alpha 2
Col5a1	1.768	0.0002	0.0114	Collagen, type V, alpha 1
Col5a2	1.601	0.0005	0.0136	Collagen, type V, alpha 2
Col5a3	1.770	0.0055	0.0451	Collagen, type V, alpha 3
Col6a1	1.741	0.0001	0.0103	Collagen, type VI, alpha 1
Col6a2	1.689	0.0001	0.0108	Collagen, type VI, alpha 2
Col6a3	1.661	0.0005	0.0138	Collagen, type VI, alpha 3
Col7a1	1.429	0.0055	0.0449	Collagen, type VII, alpha 1
Col8a1	1.780	0.0002	0.0110	Collagen, type VIII, alpha 1
Cx3cl1	1.197	0.0008	0.0166	Chemokine (C-X3-C motif) ligand 1
Cxcl16	1.365	0.0004	0.0129	Chemokine (C-X-C motif) ligand 16
Cxcr4	2.480	0.0002	0.0110	Chemokine (C-X-C motif) receptor 4
Cyr61	2.181	0.0003	0.0118	Cysteine rich angiogenic inducer 61
Dok3	2.491	0.0002	0.0110	Docking protein 3
Fam20 c	1.920	0.0001	0.0108	Family with sequence similarity 20, member C
Fbn1	1.169	0.0009	0.0171	Fibrillin 1
Fcgr3	2.168	0.0001	0.0097	Fc receptor, IgG, low affinity III
Fstl1	1.438	0.0002	0.0115	Follistatin-like 1
Gnb4	1.227	0.0006	0.0146	Guanine nucleotide binding protein (G protein), beta 4
Gpr30	1.639	0.0005	0.0142	G protein-coupled receptor 30
Gpsm3	1.804	0.0001	0.0099	G-protein signalling modulator 3 (AGS3-like, C. elegans)
H2-Q4	1.112	0.0032	0.0325	Histocompatibility 2, Q region locus 4
H2-Q6	1.565	0.0015	0.0216	Histocompatibility 2, Q region locus 6
Iqgap2	1.063	0.0005	0.0142	IQ motif containing GTPase activating protein 2
Itgal	1.178	0.0015	0.0212	Integrin alpha L
Itgam	3.776	0.0007	0.0154	Integrin alpha M
Itgax	3.934	0.0010	0.0183	Integrin alpha X
Itgb2	3.725	0.0007	0.0159	Integrin beta 2
Kng2	1.899	0.0039	0.0364	Kininogen 2
Leprel1	1.295	0.0002	0.0110	Prolyl 3-hydroxylase 2
Lilrb4	2.833	0.0024	0.0272	Leukocyte immunoglobulin like receptor B4
Mmp25	1.692	0.0005	0.0138	Matrix metallopeptidase 25
Mxra8	1.451	0.0005	0.0138	Matrix-remodelling associated 8
Nckap1 l	3.585	0.0003	0.0124	NCK associated protein 1 like
Pf4	2.367	0.0001	0.0097	Platelet factor 4
Plau	2.172	0.0003	0.0128	RAB31, member RAS oncogene family
Plaur	2.180	0.0058	0.0463	RAB3A interacting protein (rabin3)-like 1
Plod2	1.119	0.0014	0.0206	Ras association (RalGDS/AF-6) domain family member 1
Prss23	1.618	0.0002	0.0115	Protease, serine, 23
Ptafr	2.509	0.0015	0.0216	Platelet-activating factor receptor
Ptprb	1.156	0.0014	0.0209	S100 calcium binding protein A8 (calgranulin A)
Ptprj	1.864	0.0000	0.0090	Sphingosine-1-phosphate receptor 3
Rab31	1.303	0.0028	0.0300	SEC14-like 1 (S. cerevisiae)
S1pr1	1.149	0.0009	0.0179	Solute carrier family 9 (sodium/hydrogen exchanger), member 2
S1pr2	2.234	0.0001	0.0108	Solute carrier family 9 (sodium/hydrogen exchanger), member 3 regulator 2
S1pr3	1.114	0.0006	0.0146	Solute carrier family 9 (sodium/hydrogen exchanger), member 5
Sirpa	1.203	0.0006	0.0145	SplA/ryanodine receptor domain and SOCS box containing 1
Slc2a5	2.693	0.0002	0.0110	Tetratricopeptide repeat, ankyrin repeat and coiled-coil containing 1
Sparcl1	1.237	0.0040	0.0370	Transmembrane protein 158
Stk10	1.336	0.0002	0.0110	Tumour necrosis factor, alpha-induced protein 8-like 1
Tlr2	2.323	0.0009	0.0178	Tubulin, beta 6 class V
Tmc6	1.089	0.0031	0.0319	Thioredoxin reductase 3
Tmem132a	1.163	0.0022	0.0259	Ubiquitin D
Tmem173	1.912	0.0001	0.0110	Uncoupling protein 2 (mitochondrial, proton carrier)
Tnc	2.619	0.0002	0.0110	V-set and immunoglobulin domain containing 2
Tnfrsf1b	1.732	0.0001	0.0099	WT1-interacting protein

### 3.5. Construction of the Meg3 network as a ceRNA in BAT under HFD conditions

**Figure 7.**
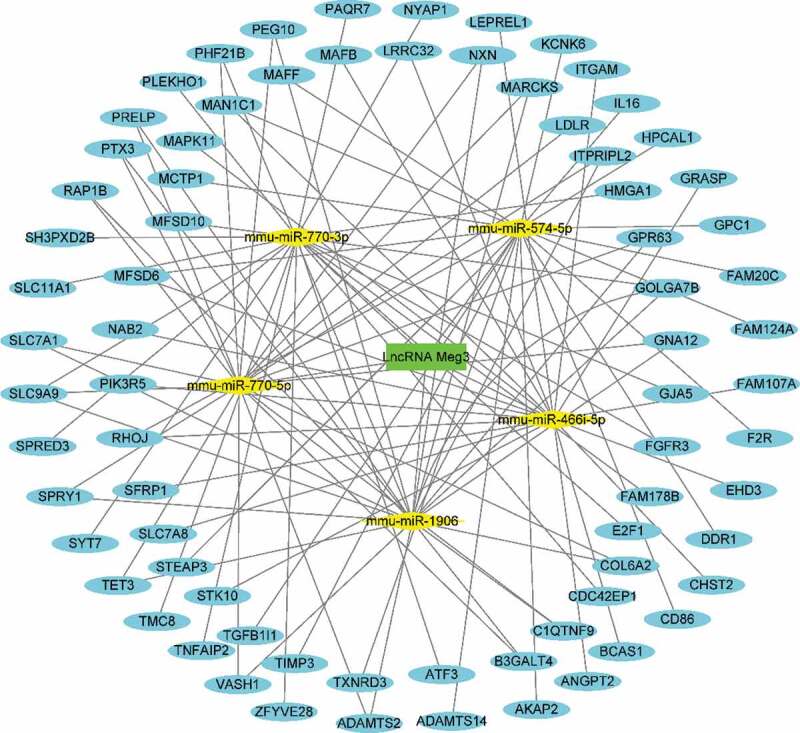
The network of *Meg3* as a ceRNA. Green indicates lncRNAs; yellow indicates miRNAs; blue indicates mRNAs

**Figure 8.**
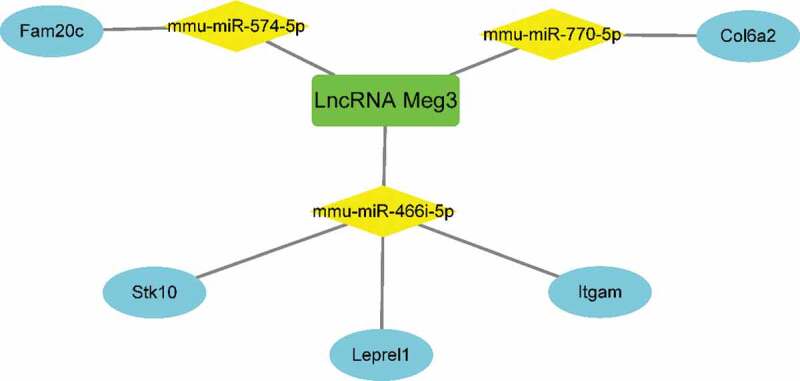
Five genes identified as hub genes modulated by *Meg3* as a ceRNA in BAT from mice on an HFD. Green indicates lncRNAs; yellow indicates miRNAs; blue indicates mRNAs

To better understand the function of DE lncRNAs on coding RNAs, *Meg3* was selected to construct a ceRNA network (Figure 7, Table 2). The overlapping genes between the most significant module of MCODE and mRNAs modulated by *Meg3*, including *Stk10, Leprel1, Itgam, Fam20 c, and Col6a2*, were identified as hub genes modulated by *Meg3* as a ceRNA in BAT under HFD conditions. *Meg3* could compete with *Stk10, Leprel1*, and *Itgam* mRNA for binding to *mmu-miR-466i-5p* miRNA; compete with *Fam20 c* mRNA for binding to *mmu-miR-574-5p*; and compete with *Col6a2* mRNA for binding to *mmu-miR-770-5p*, thus upregulating the expression of these five mRNAs in BAT under HFD conditions (Figure 8).

**Table 2.** Target miRNA prediction of lncRNA Meg3 and corresponding target mRNAs prediction

Target miRNA prediction of lncRNA Meg3	Corresponding target mRNAs prediction
mmu-miR-466i-5p	Fam107a, Pik3r5, Ehd3, Grasp, Man1c1, Angpt2, Nab2, Mapk11, Mafb, Stk10, Leprel1, Sfrp1, Mfsd6, Chst2, Slc7a8, Bcas1, Itgam, Rhoj, lrrc32
mmu-miR-574-5p	Adamts14, Mfsd6, Steap3, Fam124a, Hpcal1, Rhoj, Tgfb1i1, Ddr1, Cd86, Txnrd3, Mctp1, Gpc1, Fam20 c, Akap2, Maff, F2 r, Il16, Man1c1, Kcnk6, Timp3
mmu-miR-770-3p	Mfsd10, Golga7b, Slc11a1, Sh3pxd2b, Fam178b, Paqr7, Plekho1, Gja5, Nyap1, Slc9a9, Tmc8, E2f1, Fgfr3, Atf3, Cdc42ep1, Spred3, Hmga1, Syt7, Zfyve28, Tnfaip2
mmu-miR-770-5p	ldlr, Gpr63, Rap1b, Gna12, Col6a2, Itpripl2, Vash1, Ptx3, Prelp, Slc9a9, Phf21b, C1qtnf9, Nxn, Spry1, Adamts2, Slc7a1, Peg10, Marcks, Tet3, B3galt4
mmu-miR-1906	GOLGA7B, GPR63, RAP1B, GNA12, COL6A2, ITPRIPL2, VASH1, PTX3, PRELP, SLC9A9, PHF21B, C1QTNF9, NXN, SPRY1, ADAMTS2, SLC7A1, PEG10, MARCKS, TET3, B3GALT4, GOLGA7B

## 4. Discussion

Although the function of BAT has been demonstrated in previous studies, the effect of an HFD on the GO annotations and KEGG pathways of mRNA and lncRNA-miRNA-mRNA crosstalk in BAT remain ill-defined. In the present study, based on two microarray expression profiles analysed using bioinformatic methods, DE mRNAs and lncRNAs were identified between BAT under HFD and ND conditions. GO analysis indicated that the DE mRNAs were significantly enriched in the BP terms, cell adhesion, angiogenesis, and inflammatory response. Eguchi and colleagues reported that adipocyte adhesion molecule was implicated in adipocyte maturation and the development of obesity and thus, they speculated that cell adhesion may influence the morphology and differentiation of cells via alterations in cell signalling or cytoskeletal organization [25]. However, to the best of our knowledge, no previous studies have demonstrated cell adhesion functions in BAT. Broad-spectrum findings have reported that hypoxia plays a critical role in adipose tissue angiogenesis in response to obesity, which is rapidly induced by HFD [26–28]. This is consistent with another BP term enriched in response to hypoxia. It is noteworthy that, within the enrichment in response to hypoxia, uncoupling protein 2 (UCP2) expression was up-regulated, but UCP1 was not. UCP1 is linked to the protection against diet-induced obesity (DIO) as an integral membrane protein unique to BAT mitochondria [29]. However, UCP2 seems to have a different function, since Kim et al. demonstrated that the regulation of microglial UCP2 *in vivo* is associated with increased levels of inflammatory cytokines and that the deletion of microglial UCP2 prevents DIO [30]. According to their results and the results of the present study, we propose that UCP2 may be an inflammatory inducer in response to hypoxia in BAT under HFD conditions. Further studies are required to clarify the mechanism for the regulation of UCP2 in BAT under HFD conditions, since UCP is a promising therapeutic target for the treatment of obesity and obesity-related diseases.

In GO analysis, the significant DE mRNAs were enriched in the CC terms, membrane, cell surface, and extracellular exosome. Although exosomes derived from normal BAT can improve glucose tolerance [31,32], the functions of exosomes derived from BAT under HFD conditions are ill defined, but these exosomes are an emerging therapeutic target for metabolic diseases.

The GO analysis of MF suggested that the DE mRNAs were most significantly enriched in protein binding, which suggested that the ability of proteins to form bonds with other substances was increased. Interestingly, KEGG enrichment analysis of DE mRNAs showed that these DE mRNAs were enriched in focal adhesion, which was consistent with the GO analysis of BP, which suggested that cell adhesion may play a critical role in the BAT under HFD conditions.

The present study is the first study to demonstrate the upregulation of *Meg3* in BAT under HFD conditions and to construct the corresponding network of *Meg3* as a ceRNA (Figure 7). Increasing evidence has implicated *Meg3* in metabolic disorders. Several studies have demonstrated that *Meg3* promotes insulin resistance by serving as a ceRNA of miRNAs, to upregulate mRNA expression and consistently, *Meg3* knockdown alleviates insulin resistance in palmitate-treated hepatocytes and in mice fed an HFD [12,33]. However, You et al. demonstrated that the suppression of *Meg3* levels decreases insulin secretion from pancreatic islet beta cells [34]. Therefore, the function of *Meg3* in the development of metabolic disorders may be tissue specific. It would be of great significance to identify *Meg3* as a significant ceRNA in BAT under HFD conditions and to determine the downstream mechanism whereby *Meg3* in BAT affects the development of obesity and obesity-related diseases in response to an HFD.

In the present study, three miRNAs, including *miR-466i-5p, miR-574-5p* and *miR-770-5p* were validated as the target miRNAs of *Meg3*, predicted by the bioinformatics analysis. MiRNAs are short RNAs that can be regulated by the sponge role of lncRNA, following by modulating the expression of the downstream genes by targeting their 3′-untranslated regions (3′UTR) [35]. An increasing number of studies has demonstrated that *miR-574-5p* can be sponged by lncRNA, like lncRNA *PTCSC3* and lncRNA *MFI2-AS1*, to modulate growth and metastasis of cancer cells [36,37]. In addition, miR-770-5p has been found to be downregulated by lncRNA *TPT1-AS1* and correspondingly upregulated the expression of STMN1, leading to promotion of the proliferation of Glioma cells [38].

Interestingly, the genes targeted by *miR-466i-5p, miR-574-5p* and *miR-770-5p*, were identified as hub genes modulated by *Meg3* as a ceRNA in BAT under HFD conditions, including *Stk10, Leprel1, Itgam, Fam20 c, and Col6a2*. Among them, *Leprel1, Itgam* and *Col6a2* are expressed in adipose tissues and involved in the regulation of inflammation, fibrosis, insulin signalling and so on, respectively [39–41]. There are no findings linking *Stk10* and *Fam20 c* with adipocytes or adipose tissues, although *Stk10* possesses anti-apoptotic property [42,43] and *Fam20 c* is associated with insulin production in pancreases β cells [44]. Furthermore, to date, there are no previous reports correlating these five genes with *Meg3*. Further studies are thus required to validate the correlation between *Meg3* and the five hub genes identified in the present study and to determine the mechanisms whereby *Meg3* and these five hub genes are involved in metabolic processes and the development of metabolic disorders.

Taken together, the present study, for the first time, identifies changes in both mRNA and lncRNA levels contributing to HFD-induced obesity and indicates that lncRNA *Meg3* might modulate several mRNAs *via* binding to microRNAs competitively. However, this study is a purely bioinformatics analysis aiming to help investigators to explore the DE RNAs and DE LncRNAs in brown adipose tissue in mice fed with HFD. The conclusions are required to be verified by *in vitro* and *in vivo* experimental settings closer to the clinical reality or in the invidious with obesity or diabetes. Only in this case can we better understand the exact functions of *Meg3* and its five downstream genes in human BAT and also uncover mechanisms related to the development and progression of obesity and diabetes.

## Supplementary Material

Supplemental Material

Supplemental Material

## References

[1] Rabot S, Membrez M, Blancher F, et al. High fat diet drives obesity regardless the composition of gut microbiota in mice. Sci Rep. 2016;6:32484. 27577172 10.1038/srep32484 PMC5006052

[2] Riobo Servan P. Obesity and diabetes. Nutr Hosp. 2013;28(Suppl 5):138–143. 24010754 10.3305/nh.2013.28.sup5.6929

[3] Bhupathiraju SN, Hu FB. Epidemiology of obesity and diabetes and their cardiovascular complications. Circ Res. 2016;118(11):1723–1735. 27230638 10.1161/CIRCRESAHA.115.306825 PMC4887150

[4] Ghaben AL, Scherer PE. Adipogenesis and metabolic health. Nat Rev Mol Cell Biol. 2019;20(4):242–258. 30610207 10.1038/s41580-018-0093-z

[5] Fedorenko A, Lishko PV, Kirichok Y. Mechanism of fatty-acid-dependent UCP1 uncoupling in brown fat mitochondria. Cell. 2012;151(2):400–413. 23063128 10.1016/j.cell.2012.09.010 PMC3782081

[6] Cypess AM, Kahn CR. Brown fat as a therapy for obesity and diabetes. Curr Opin Endocrinol Diabetes Obes. 2010;17(2):143–149. 20160646 10.1097/MED.0b013e328337a81f PMC3593105

[7] Harms M, Seale P. Brown and beige fat: development, function and therapeutic potential. Nat Med. 2013;19(10):1252–1263. 24100998 10.1038/nm.3361

[8] Heck AM, Wilusz J. The Interplay between the RNA decay and translation machinery in eukaryotes. Cold Spring Harb Perspect Biol. 2018;10(5):a032839. 10.1101/cshperspect.a032839 PMC5932591 29311343

[9] Kung JT, Colognori D, Lee JT. Long noncoding RNAs: past, present, and future. Genetics. 2013;193(3):651–669. 23463798 10.1534/genetics.112.146704 PMC3583990

[10] Salmena L, Poliseno L, Tay Y, et al. A ceRNA hypothesis: the Rosetta stone of a hidden RNA language? Cell. 2011;146(3):353–358. 21802130 10.1016/j.cell.2011.07.014 PMC3235919

[11] Zhu X, Li H, Wu Y, et al. CREB-upregulated lncRNA MEG3 promotes hepatic gluconeogenesis by regulating miR-302a-3p-CRTC2 axis. J Cell Biochem. 2019;120(3):4192–4202. 30260029 10.1002/jcb.27706

[12] Zhu X, Li H, Wu Y, et al. lncRNA MEG3 promotes hepatic insulin resistance by serving as a competing endogenous RNA of miR-214 to regulate ATF4 expression. Int J Mol Med. 2019;43(1):345–357. 30431065 10.3892/ijmm.2018.3975 PMC6257836

[13] Chen Y, Zhang Z, Zhu D, et al. Long non-coding RNA MEG3 serves as a ceRNA for microRNA-145 to induce apoptosis of AC16 cardiomyocytes under high glucose condition. Biosci Rep. 2019;39(6). DOI:10.1042/BSR20190444 PMC6554216 31085717

[14] Shi F, Collins S. Second messenger signaling mechanisms of the brown adipocyte thermogenic program: an integrative perspective. Horm Mol Biol Clin Investig. 2017;31(2). 10.1515/hmbci-2017-0062 28949928

[15] Zhao XY, Li S, Wang GX, et al. A long noncoding RNA transcriptional regulatory circuit drives thermogenic adipocyte differentiation. Mol Cell. 2014;55(3):372–382. 25002143 10.1016/j.molcel.2014.06.004 PMC4127104

[16] Clough E, Barrett T. The gene expression omnibus database. Methods Mol Biol. 2016;1418:93–110. 27008011 10.1007/978-1-4939-3578-9_5 PMC4944384

[17] Lo KA, Huang S, Walet ACE, et al. Adipocyte long-noncoding RNA transcriptome analysis of Obese mice identified Lnc-leptin, which regulates leptin. Diabetes. 2018;67(6):1045–1056. 29519872 10.2337/db17-0526

[18] Schmidt E, Dhaouadi I, Gaziano I, et al. LincRNA H19 protects from dietary obesity by constraining expression of monoallelic genes in brown fat. Nat Commun. 2018;9(1):3622. 30190464 10.1038/s41467-018-05933-8 PMC6127097

[19] Leek JT, Johnson WE, Parker HS, et al. The sva package for removing batch effects and other unwanted variation in high-throughput experiments. Bioinformatics. 2012;28(6):882–883. 22257669 10.1093/bioinformatics/bts034 PMC3307112

[20] Ritchie ME, Phipson B, Wu D, et al. limma powers differential expression analyses for RNA-sequencing and microarray studies. Nucleic Acids Res. 2015;43(7):e47. 25605792 10.1093/nar/gkv007 PMC4402510

[21] Khomtchouk BB, Van Booven DJ, Wahlestedt C. HeatmapGenerator: high performance RNAseq and microarray visualization software suite to examine differential gene expression levels using an R and C++ hybrid computational pipeline. Source Code Biol Med. 2014;9(1):30. 25550709 10.1186/s13029-014-0030-2 PMC4279803

[22] da Huang W, Sherman BT, Lempicki RA. Bioinformatics enrichment tools: paths toward the comprehensive functional analysis of large gene lists. Nucleic Acids Res. 2009;37(1):1–13. 19033363 10.1093/nar/gkn923 PMC2615629

[23] Franceschini A, Szklarczyk D, Frankild S, et al. STRING v9.1: protein-protein interaction networks, with increased coverage and integration. Nucleic Acids Res. 2013;41(Database issue):D808–15. 23203871 10.1093/nar/gks1094 PMC3531103

[24] Bandettini WP, Kellman P, Mancini C, et al. MultiContrast Delayed Enhancement (MCODE) improves detection of subendocardial myocardial infarction by late gadolinium enhancement cardiovascular magnetic resonance: a clinical validation study. J Cardiovasc Magn Reson. 2012;14:83. 23199362 10.1186/1532-429X-14-83 PMC3552709

[25] Eguchi J, Wada J, Hida K, et al. Identification of adipocyte adhesion molecule (ACAM), a novel CTX gene family, implicated in adipocyte maturation and development of obesity. Biochem J. 2005;387(Pt 2):343–353. 15563274 10.1042/BJ20041709 PMC1134962

[26] Disanzo BL, You T. Effects of exercise training on indicators of adipose tissue angiogenesis and hypoxia in obese rats. Metabolism. 2014;63(4):452–455. 24412283 10.1016/j.metabol.2013.12.004

[27] Xue Y, Petrovic N, Cao R, et al. Hypoxia-independent angiogenesis in adipose tissues during cold acclimation. Cell Metab. 2009;9(1):99–109. 19117550 10.1016/j.cmet.2008.11.009

[28] Xie Q, Xie J, Zhong J, et al. Hypoxia enhances angiogenesis in an adipose-derived stromal cell/endothelial cell co-culture 3D gel model. Cell Prolif. 2016;49(2):236–245. 26997164 10.1111/cpr.12244 PMC6496300

[29] Wang H, Liu L, Lin JZ, et al. Browning of white adipose tissue with roscovitine induces a distinct population of UCP1(+) adipocytes. Cell Metab. 2016;24(6):835–847. 27974179 10.1016/j.cmet.2016.10.005 PMC6674884

[30] Kim JD, Yoon NA, Jin S, et al. Microglial UCP2 mediates inflammation and obesity induced by high-fat feeding. Cell Metab. 2019;30:952–962.e5. 10.1016/j.cmet.2019.08.010 PMC7251564 31495690

[31] Thomou T, Mori MA, Dreyfuss JM, et al. Adipose-derived circulating miRNAs regulate gene expression in other tissues. Nature. 2017;542(7642):450–455. 28199304 10.1038/nature21365 PMC5330251

[32] Chen Y, Pfeifer A. Brown fat-derived exosomes: small vesicles with big impact. Cell Metab. 2017;25(4):759–760. 28380368 10.1016/j.cmet.2017.03.012

[33] Chen DL, Shen DY, Han CK, et al. LncRNA MEG3 aggravates palmitate-induced insulin resistance by regulating miR-185-5p/Egr2 axis in hepatic cells. Eur Rev Med Pharmacol Sci. 2019;23(12):5456–5467. 31298399 10.26355/eurrev_201906_18215

[34] You L, Wang N, Yin D, et al. Downregulation of long noncoding RNA Meg3 affects insulin synthesis and secretion in mouse pancreatic beta cells. J Cell Physiol. 2016;231(4):852–862. 26313443 10.1002/jcp.25175

[35] Olgun G, Sahin O, Tastan O. Discovering lncRNA mediated sponge interactions in breast cancer molecular subtypes. BMC Genomics. 2018;19(1):650. 30180792 10.1186/s12864-018-5006-1 PMC6122485

[36] Tong R, Zhang J, Wang C, et al. LncRNA PTCSC3 inhibits the proliferation, invasion and migration of cervical cancer cells via sponging miR-574-5p. Clin Exp Pharmacol Physiol. 2020;47(3):439–448. 31587336 10.1111/1440-1681.13186

[37] Li C, Tan F, Pei Q, et al. Non-coding RNA MFI2-AS1 promotes colorectal cancer cell proliferation, migration and invasion through miR-574-5p/MYCBP axis. Cell Prolif. 2019;52(4):e12632. 31094023 10.1111/cpr.12632 PMC6668983

[38] Jia L, Song Y, Mu L, et al. Long noncoding RNA TPT1-AS1 downregulates the microRNA-770-5p expression to inhibit glioma cell autophagy and promote proliferation through STMN1 upregulation. J Cell Physiol. 2020;235(4):3679–3689. 31637705 10.1002/jcp.29262 PMC13482729

[39] Jarnum S, Kjellman C, Darabi A, et al. LEPREL1, a novel ER and Golgi resident member of the Leprecan family. Biochem Biophys Res Commun. 2004;317(2):342–351. 15063763 10.1016/j.bbrc.2004.03.060

[40] Kwon EY, Shin SK, Cho YY, et al. Time-course microarrays reveal early activation of the immune transcriptome and adipokine dysregulation leads to fibrosis in visceral adipose depots during diet-induced obesity. BMC Genomics. 2012;13:450. 22947075 10.1186/1471-2164-13-450 PMC3447724

[41] Sun K, Park J, Gupta OT, et al. Endotrophin triggers adipose tissue fibrosis and metabolic dysfunction. Nat Commun. 2014;5:3485. 24647224 10.1038/ncomms4485 PMC4076823

[42] Fukumura K, Yamashita Y, Kawazu M, et al. STK10 missense mutations associated with anti-apoptotic function. Oncol Rep. 2013;30(4):1542–1548. 23842845 10.3892/or.2013.2605

[43] Arora S, Gonzales IM, Hagelstrom RT, et al. RNAi phenotype profiling of kinases identifies potential therapeutic targets in Ewing’s sarcoma. Mol Cancer. 2010;9:218. 20718987 10.1186/1476-4598-9-218 PMC2933621

[44] Kang T, Boland BB, Alarcon C, et al. Proteomic analysis of restored insulin production and trafficking in obese diabetic mouse pancreatic islets following euglycemia. J Proteome Res. 2019;18(9):3245–3258. 31317746 10.1021/acs.jproteome.9b00160

