# Supplementary material for: Identification of differentially expressed mRNA and the Hub mRNAs modulated by lncRNA Meg3 as a competing endogenous RNA in brown adipose tissue of mice on a high-fat diet
Source: Adipocyte. 2020 Jul 2;9(1):347–59. doi: 10.1080/21623945.2020.1789283 (PMC7469684; doi:10.1080/21623945.2020.1789283)
Supplement: Supplemental Material [file KADI_A_1789283_SM8986.docx]

**Table S2:** The top 10 upregulated expression lncRNA, including lncRNA Meg3

| **ID** | **logFC** | **P.Value** | **adj.P.Val** |
| --- | --- | --- | --- |
| Gm5627 | 3.218 | 0.0007 | 0.0158 |
| Gm19705 | 2.214 | 0.0004 | 0.0133 |
| Gm11730 | 2.187 | 0.0016 | 0.0220 |
| 2810032G03Rik | 2.176 | 0.0000 | 0.0047 |
| Gm21917 | 2.148 | 0.0026 | 0.0283 |
| Meg3 | 2.029 | 0.0000 | 0.0074 |
| A730081D07Rik | 2.028 | 0.0001 | 0.0108 |
| 6030445D17Rik | 2.004 | 0.0001 | 0.0107 |
| Gm16091 | 1.936 | 0.0002 | 0.0110 |
| 2810430I11Rik | 1.908 | 0.0017 | 0.0226 |
